# Management of primary blast lung injury: a comparison of airway pressure release versus low tidal volume ventilation

**DOI:** 10.1186/s40635-020-00314-2

**Published:** 2020-06-23

**Authors:** Timothy E. Scott, Anup Das, Mainul Haque, Declan G. Bates, Jonathan G. Hardman

**Affiliations:** 1 Academic Department of Military Anaesthesia and Critical Care, Royal Centre for Defence Medicine, ICT Centre, Birmingham, B15 2SQ UK; 2 School of Engineering, University of Warwick, Coventry, CV4 7AL UK; 3 School of Mathematics and Physics, University of Portsmouth, Portsmouth, UK; 4 Anaesthesia & Critical Care, Division of Clinical Neuroscience, School of Medicine, University of Nottingham, Nottingham, NG7 2UH UK; 5 Nottingham University Hospital NHS Trust, Nottingham, NG7 2UH UK

**Keywords:** Primary blast lung injury, Acute respiratory distress syndrome, Low tidal volume ventilation, Airway pressure release ventilation, Ventilator-induced lung injury, Computational modelling

## Abstract

**Background:**

Primary blast lung injury (PBLI) presents as a syndrome of respiratory distress and haemoptysis resulting from explosive shock wave exposure and is a frequent cause of mortality and morbidity in both military conflicts and terrorist attacks. The optimal mode of mechanical ventilation for managing PBLI is not currently known, and clinical trials in humans are impossible due to the sporadic and violent nature of the disease.

**Methods:**

A high-fidelity multi-organ computational simulator of PBLI pathophysiology was configured to replicate data from 14 PBLI casualties from the conflict in Afghanistan. Adaptive and responsive ventilatory protocols implementing low tidal volume (LTV) ventilation and airway pressure release ventilation (APRV) were applied to each simulated patient for 24 h, allowing direct quantitative comparison of their effects on gas exchange, ventilatory parameters, haemodynamics, extravascular lung water and indices of ventilator-induced lung injury.

**Results:**

The simulated patients responded well to both ventilation strategies. Post 24-h investigation period, the APRV arm had similar PF ratios (137 mmHg vs 157 mmHg), lower sub-injury threshold levels of mechanical power (11.9 J/min vs 20.7 J/min) and lower levels of extravascular lung water (501 ml vs 600 ml) compared to conventional LTV. Driving pressure was higher in the APRV group (11.9 cmH_2_O vs 8.6 cmH_2_O), but still significantly less than levels associated with increased mortality.

**Conclusions:**

Appropriate use of APRV may offer casualties with PBLI important mortality-related benefits and should be considered for management of this challenging patient group.

## Background

Primary blast lung injury (PBLI) results from explosive shock wave exposure and is a frequent cause of mortality and morbidity in both military conflicts and acts of terrorism [1]. It is particularly associated with explosions in confined spaces, such as terrorist attacks on public transportation systems [2]. Defined as “acute lung injury within 12 h of blast exposure which is not due to penetrating or blunt injury” [3], it presents as a syndrome of respiratory distress and haemoptysis and is frequently complicated by pneumothoraces. Severe cases progress as acute respiratory distress syndrome (ARDS). The majority of casualties with the condition will require ventilatory support in an intensive care unit (ICU) [4] and so it can generate unpredictable and significant demands on medical resources. As PBLI is by nature a sporadic disease born out of conflict, randomized controlled clinical trials to investigate alternative management strategies are not feasible—clinical care has been, and will continue to be, guided by surrogate models of the disease. Such models have traditionally been in vivo animal models [5–7], but there has recently been increasing interest in the use of in silico models of human PBLI pathophysiology [8–10].

Currently, the globally recognised best practice for mechanical ventilation in casualties with acute lung injury (including PBLI) is the approach advocated by the ARDS network (ARDSnet) group [11]. The ARDSnet “protective ventilation” approach utilises low tidal volumes (LTV) and relatively high positive end-expiratory pressure (PEEP) to minimise mechanical volutrauma and limit atelectasis. It advocates using the lowest acceptable inspired concentration of oxygen to prevent oxygen toxicity and tolerates hypercapnia within the limits of cardiovascular stability (assuming the patient has not suffered a head injury).

Airway pressure release ventilation (APRV) is an alternative mode of ventilation which is also currently in use for the management of hypoxic respiratory failure including ARDS in mechanically ventilated patients [12]. APRV is widely available on existing mechanical ventilators and may be adopted as a second-line therapy if ventilatory parameters continue to deteriorate despite LTV. It consists of the application of continuous positive airway pressure with scheduled, short, intermittent pressure releases that facilitate ventilation of carbon dioxide but are too brief to allow alveolar collapse and thus atelectasis [13–15].

Recent work utilising a porcine model of sepsis-induced ARDS compared LTV ventilation with APRV [16, 17]. The results of this study suggested that APRV was potentially superior to LTV in a number of significant ways. In particular, APRV prevented clinical and histological lung injury by preserving alveolar epithelial integrity, reducing lung oedema, preserving pulmonary surfactant and maintaining alveolar stability. In a recent clinical trial involving 138 human subjects, early application of APRV in patients with ARDS improved oxygenation and respiratory system compliance, decreased plateau airway pressures and reduced the duration of both mechanical ventilation and ICU stay, compared with LTV [18]. However, significant uncertainty still exists regarding the precise mode of action of APRV, optimal ventilator settings for its implementation, and its effect on key VILI indicators such as driving pressure [19].

The optimal mode of mechanical ventilation in PBLI is not currently known as this is the first work of this type. However, if we extrapolate findings from the ARDSnet trial (which was open to all causes of severe acute lung injury), we can postulate that reducing inspiratory plateau pressures, inspired concentration of oxygen, extravascular lung water (EVLW) and ventilator-induced lung injury (VILI) may result in improved patient outcomes. In this study, we employ a high-fidelity computational simulator, trained to replicate data from 14 real human PBLI casualties, to compare the efficacy of APRV and LTV ventilation over the first 24 h following injury.

## Methods

Our PBLI simulator is a high-fidelity iterative computational model of human cardiopulmonary pathophysiology [10]. It can accommodate both mechanical and spontaneous ventilation, evolving acute lung injury including non-cardiogenic pulmonary oedema as well as ventilator-induced lung injury (VILI). The simulator can be calibrated to match individual patient data using a high-performance computing cluster (eight servers, each with 2 x ten-core Intel Xeon processors with 128 GB RAM) based at the University of Warwick. The model had previously been shown to accurately predict the cardiorespiratory effects of moderate to severe primary blast lung injury in a large animal model [20–22]. It has been validated against human cardiopulmonary physiology [23] and is used in a broad range of clinical research [24–27]. In the absence of medical intervention, application of PBLI in the simulator produces the same natural history detailed in the animal modelling referenced above. A detailed description of the mathematical principles and equations underlying the simulator is available in the online data supplement.

For this study, we generated a bank of 14 in silico human casualties to act as virtual study subjects. A detailed clinical database describing 14 military casualties with PBLI was used to re-create each replica casualty [28]. Clinical data consisted of arterial blood gas and ventilator settings recorded at irregular time points throughout the patients’ intensive care unit admission. From this clinical data, single time points were selected for each patient based on the following criteria: (1) the time point was recorded at the earliest possible moment after hospital admission, and (2) the dataset across the arterial blood gas values and ventilator settings was most complete. The PBLI computational model was then calibrated using advanced global optimisation algorithms to accurately replicate the pathophysiological conditions of each individual patient at these time points. This calibration process involves finding a distinct combination of model parameters that reproduce the clinical data most accurately. The model parameters include airway resistances, alveolar compliances, vascular resistances, and permeabilities of the alveolar membranes.

According to the Berlin definition [29], seven of our casualties had severe ARDS and seven had moderate ARDS. The 14 in silico subjects are assumed to have no other injury, be euvolaemic and weigh 70 kg. We also assume that any pneumothoraces have been adequately drained. Table 1 gives the baseline settings of the patients.

**Table 1:** Baseline characteristics of patients

Parameters	Mean	Std
FiO_2_	0.67	0.2
Tidal volume, ml	580	94
PEEP, cmH_2_O	6	1.8
Vent rate, bpm	16	3.0
Duty cycle	0.44	0.1
PaO_2_, kPa	13.49	7.4
PaCO_2_, kPa	5.83	1.0
PF ratio, mmHg	171	113
SpO_2_, %	92	7.6
pH	7.35	0.1
Base excess, mmol/l	-2.05	3.5
Haemoglobin, g/dl	11.5	2.4
Mean art. pressure, mmHg	95	14
Heart rate, bpm	93	9
Mean pulmonary art. pressure, mmHg	20	4
Cardiac output, l/min	5.4	0.7
EVLW, ml	708	155

One hour following injury (replicating the time required to reach a hospital and undergo initial casualty management), each subject was modelled for 24 h in each of the two ventilatory modes studied. Flexible, responsive and clinically realistic ventilation strategies were implemented for each mode, as described in Table 2. Ventilator settings were adjusted on an hourly basis following interrogation of current in silico arterial blood gas values with the aim of achieving an arterial partial pressure of oxygen of 9.0 kPa (67.5 mmHg). The simulator applied constant pressure generated mechanical ventilation when applying APRV and constant flow generated mechanical ventilation when applying ARDSnet ventilation. The simulations are performed with a time step of 10 ms. Measurements of key pulmonary, ventilatory and cardiovascular physiological parameters were made at one hourly intervals. Additionally, driving pressure (Δ*P*) and mechanical power (*MP*) were also calculated at one hourly intervals. These novel indices are independent, dynamic and patient-centric targets for reducing VILI (and potentially mortality) in ARDS [30–32]. Driving pressure (Δ*P*) is defined as the difference between plateau pressure (*P*_*plat*_) and the total positive end expiratory pressure (*PEEPt)*:

1 $$ \Delta P= Pplat- PEEPt $$

**Table 2:** Ventilatory protocol applied by the simulator over the 24-h duration of the study

Ventilatory parameters	ARDSnet (LTV)	APRV
Initial settings	Tidal volume of 6 ms/kg Respiratory rate 16 bpm PEEP 10 cm H_2_0 Plateau pressure limited to 30 cmH_2_O FiO_2_ 50% I:E ratio 1:1	P_High_–25 cmH_2_O (20–30 cmH_2_O) P_Low_–0 cmH_2_O T_High_–5 s (4–6) T_Low_–0.5 s (0.35–0.6)* P_High_ limited to 30 cmH_2_O FiO_2_ 50%
One hourly adjustments	If PaO_2_ < 9.0 kPa, increase FiO_2_ along scale below. If PaO_2_ > 10.0 kPa, decrease FiO_2_ along scale below. Minimum FiO_2_–30%	If PaO_2_ < 9.0 kPa, increase FiO_2_ along the scale below and increase P_High_ by 1 cmH_2_O. If pressure limit is reached, increase T_High_ by 0.1 s. If PaO_2_ > 10.0 kPa, decrease FiO_2_ along scale below and decrease P_High_ by 1 cmH_2_O. If P_High_ is 20 cmH_2_O or less, decrease T_High_ by 0.1 s.
FiO_2_ scale	0.3 0.4 0.5 0.6 0.7 0.8 0.9 1.0	0.3 0.4 0.5 0.6 0.7 0.8 0.9 1.0
FiO_2_-based adjustments	FiO_2_ 0.3 0.4 0.5 0.6 0.7 0.8 0.9 1.0 PEEP 6 8 10 12 14 16 18 20	
pH-based adjustments**	If pH is 7.25 or less, increase respiratory rate by 2 (up to 34 bpm). If pH is 7.5 or greater, decrease respiratory by 2 (down to 12)	If pH is 7.25 or less, increase P_High_ by 1 cmH_2_O. If P_High_ ≥ 25 and T_high_ < 6, increase T_High_ by 0.1 s. If pH ≥ 7.5 – if P_High_ > 20, decrease P_High_ by 1 cmH_2_O. If P_High_ ≥ 25 and T_High_ ≥ 5.5, decrease T_High_ by 0.1 s.

*T low will approximate to 75% peak-expiratory flow rate

**If casualty pH deviates above 7.5, an alkalotic management strategy will be introduced to the protocol

The value of *P*_*plat*_ is calculated directly from the simulator and represents the end-inspiratory lung pressure. The *PEEPt* is taken as the end-expiratory lung pressure. A value of Δ*P* of 15 cmH_2_O or greater is strongly associated with mortality [33].

Mechanical power is an index that attempts to describe the total energy delivered to the lungs by the ventilator and is calculated as:

2 $$ MP=0.098\times VR\times {V}_T\times \left({P}_{peak}-0.5\times \Delta  P\right) $$

where *VR* is the ventilator set respiratory rate, *V*_*T*_ is the tidal volume and *P*_*peak*_ is the peak inspiratory pressure. Values of *MP* greater than 22 J/min have been shown to independently predict VILI and increased mortality [34]. All data are presented as mean (± SE).

## Results

**Fig. 1:**
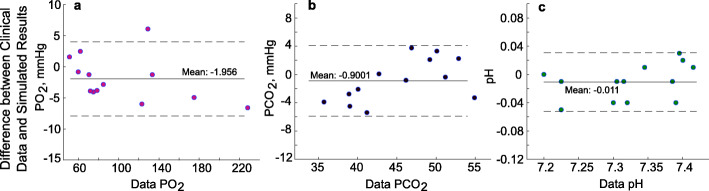
Bland Altman plots for simulator outputs with reference to data. Mean is represented by the solid line, while the dashed lines indicate ± 1.96 standard deviation from the mean

Figure 1 shows the results of model calibration to values obtained from data for arterial partial pressures of oxygen and carbon dioxide (PaO_2_, PaCO_2_) and arterial pH, as Bland Altman plots. The model was also calibrated to nominal values of cardiac output (CO), extravascular lung water (EVLW), mean arterial pressure (MAP) and mean pulmonary arterial pressures (MPAP) at the same time. These additional parameters are recorded in Table 1. The physiology of one of the in vivo casualties was too deranged to model. Casualty number 9 had initial PaCO_2_ values ranging from 12.0 to 14.3 kPa over the first 4 h of hospital treatment, and in the absence of a clinical explanation, the simulator was unable to replicate the data. The remaining 13 in silico casualties completed 24 h of ventilatory modelling with each ventilatory mode. An alkalosis management strategy to correct a pH of greater than 7.5 was not required.

### Effect of ventilation mode on gas exchange

**Fig. 2:**
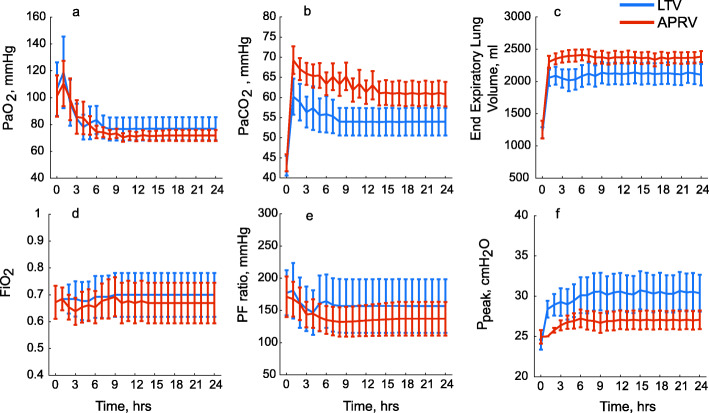
Graphical results for hourly changes in arterial partial pressures of **a** oxygen, **b** carbon dioxide, **c** end expiratory lung volume, **d** inspired concentration of oxygen, **e** PF ratios, and **f** peak ventilatory pressures (P_peak_). The LTV arm is represented in blue and the APRV arm is in red

Figure 2 shows the hourly results for inspired concentration of oxygen (FiO_2_), PaO_2_, PaCO_2_ and PF ratios (PaO_2_/FiO_2_). Values for both FiO_2_ and PaO_2_ stabilise by approximately hour 12 of the study and then remain static for the remainder of the trial. By the end of 24 h, a mean FiO_2_ of 0.70 (± 0.08) and 0.67(± 0.08) was applied, achieving a mean PaO_2_ of 10.24 (± 1.14) kPa and 9.58 (± 0.54) kPa in the LTV and APRV arms, respectively. This mean value is skewed above the trial target value of achieving a PaO_2_ of 9.0 kPa by two moderate cases responding very well to mechanical ventilation on the lowest FiO_2_ permitted by our ventilatory protocol (0.3). PaCO_2_ in the LTV arm remains lower than that seen in the APRV arm throughout the study with final mean values of 7.20 (± 0.45) kPa and 8.11 (± 0.40) kPa, respectively. PF ratios diverge and stabilise after approximately 10 h of mechanical ventilation with final mean values of 157 (± 42) mmHg and 137 (± 26) mmHg for LTV and APRV, respectively.

### Effect of ventilation mode on ventilatory parameters

Figure 2 also shows the hourly results for end-expiratory lung volume (EELV) and peak ventilatory pressures. Predictably, EELV quickly increases in our casualties once they are mechanically ventilated. Values then remain relatively constant throughout the remaining 24-hour period. Casualties in the LTV arm had a mean EELV of 2103 (± 165) ml at the end of the trial compared to a mean EELV of 2385 (± 85) ml in the APRV group. Peak airway pressure in the LTV group remains slightly higher than that seen in the APRV group throughout the trial period with mean values of 30.4 (± 2.2) cmH_2_O and 27.1 (± 1.2) cmH_2_O, respectively. As dictated by our ventilatory protocol, mean tidal volume in the LTV group is 420 (± 2.79) ml (approximating to 6 ml/kg) at the end of the trial.

### Effect of ventilation mode on haemodynamics

**Fig. 3:**
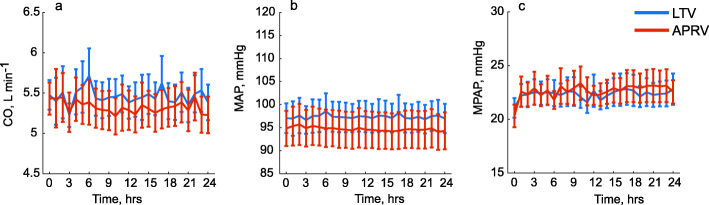
Graphical results for hourly changes in **a** cardiac output (CO), **b** mean arterial pressure (MAP), and **c** mean pulmonary arterial pressure (MPAP). The LTV arm is represented in blue and the APRV arm is in red

Figure 3 demonstrates the course of mean arterial pressure, cardiac output and mean pulmonary artery pressure during the study. Mean arterial pressures are relatively constant in both modes of ventilation with final mean values of 97.0 (± 3.1) mmHg in the LTV group and 94.3 (± 4.0) mmHg in the APRV group. Similarly, there is little difference in mean pulmonary arterial pressure with final mean values of 22.8 (± 1.5) mmHg for LTV and 22.6 (± 1.1) mmHg for APRV. Mean cardiac output at the end of the trial is 5.38 (± 0.22) l/min and 5.23 (± 0.23) l/min in the LTV and APRV arms, respectively.

### Effect of ventilation mode on VILI indices and extravascular lung water

**Fig. 4:**
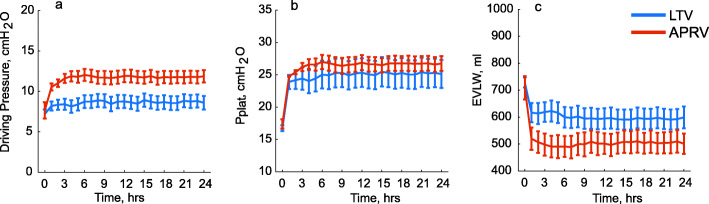
Graphical results for hourly changes in key indices of ventilator-induced lung injury: **a** driving pressures, **b** plateau pressures (P_plat_), and **c** extravascular lung water (EVLW). The LTV arm is represented in blue and the APRV arm is in red

**Fig. 5:**
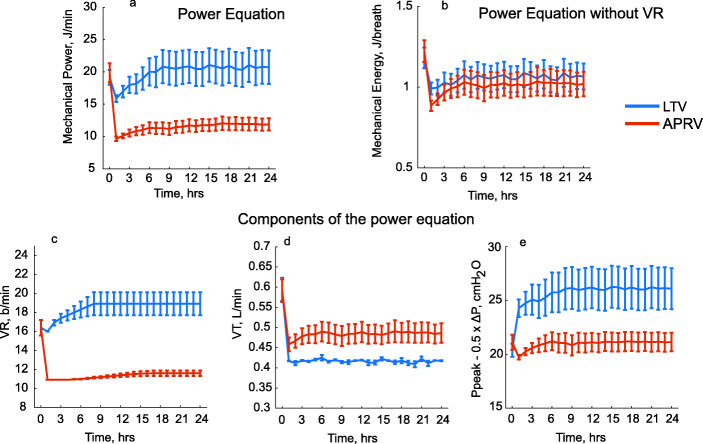
Graphical results for hourly changes in **a** mechanical power and **b** mechanical power without the ventilatory rate component. The figures also show hourly changes in the main components of the power equation (Eq. 2 in the text), including **c** ventilatory rate (VR), **d** tidal volume (VT) and **e** the pressure component. The LTV arm is represented in blue and the APRV arm is in red

Figure 4 shows the hourly results for driving pressure, plateau pressures and extravascular lung water. The driving pressure increases to a steady state mean value of 8.6 (± 0.85) cmH_2_O and 11.9 (± 0.8) cmH_2_O in LTV and APRV, respectively, within 8 h of mechanical ventilation. The mean plateau pressure in the LTV group is 25.1 (± 2.2) cmH_2_O. Predictably, extravascular lung water decreases in both arms of the study once mechanical ventilation is initiated and again reaches a stable value following approximately 8 h of mechanical ventilation. At 24 h, the mean extravascular lung water content in the LTV group is 600 (± 41) ml and 501 (± 38) ml for the APRV group. Figure 5 shows the changes in ventilator-delivered mechanical power and its constituent parts, including the *VR*, *V*_*T*_ and the pressure component *P*_*peak*_ − 0.5 × *∆P*, separately. Figure 5 also displays the mechanical energy delivered by the ventilator in a single breath, calculated by removing *VR* from the power equation (Fig. 5b). Final values of mechanical power were calculated to be 20.7 (± 2.6) J/min for LTV and 11.9 (± 1.0) J/min in APRV.

## Discussion

This study presents an examination of the potential outcomes for a cohort of patients suffering PBLI managed with two different ventilatory strategies during their first 24 h of mechanical ventilation. Our ventilatory protocol proved to be a reliable and robust decision-making framework. It achieved stable ventilated casualties within approximately 8–10 h despite significant lung injury in most cases.

The study suggests a good response to both LTV-directed mechanical ventilation and APRV in young otherwise healthy adults with PBLI. Epidemiological data gathered following the conflict in Afghanistan demonstrates a good response to LTV in young adult males with PBLI. This response is replicated in this modelling study. APRV also performs well and achieves the target arterial oxygenation and greater functional residual capacity implying reduced atelectasis. Our most important mortality-related outcomes are driving pressure and mechanical power. APRV applied a greater driving pressure than LTV throughout the study; however, in this cohort of patients, driving pressure in both modes of ventilation remained comfortably below the 15 cmH_2_O ceiling above which mortality is predicted to increase. Applied mechanical power, on the other hand, is significantly lower in the APRV group. Patients ventilated with APRV experienced lower levels of applied mechanical power to the respiratory system, with all the patients in the APRV arm below the 22 J/min threshold for increased mortality. In comparison, 4 out of 13 patients in the LTV arm breached this threshold. As an index of risk for VILI, the concept of mechanical power is of growing interest within the clinical community [35, 36]. Our results indicate that LTV delivers a higher mechanical power to the lung than APRV (20.7 J/min vs 11.9 J/min). A closer examination of the component parts of the mechanical power applied by the ventilator to our casualties (Fig. 5 c–e) reveals that the mechanical ventilatory rate required in the APRV cohort was consistently less than in the LTV group. Removing the effect of ventilatory rate from the mechanical power calculation (as seen in Fig. 5b), we see that in both modes of ventilation, the energy delivered in a breath then approximates in value. This highlights the importance of ventilatory rate as a factor in the mechanical power delivered to the respiratory system during mechanical ventilation.

While APRV performs as well as, and in some important respects better than LTV in this study, the clinically and statistically significant benefits relating to oxygenation that have been demonstrated in animal models of acute lung injury were not seen [16, 17]. Note, however, that such models use sepsis or applied chemical injury to induce acute lung injury. Sepsis (or other ongoing inflammatory processes) produce a persisting systemic epithelial failure and inflammatory cascade unlike the more geographic injury seen in primary blast lung injury. Indeed, trauma-related acute lung injury is recognised as a milder and more responsive disease compared to non-trauma-related acute lung injury [37, 38]. Thus, we can expect that differences related to oxygenation between effective treatments will be more marginal in PBLI. Additionally, these animal trials pre-date our understanding of the importance of driving pressure and applied mechanical power as predictors of VILI and patient outcomes. Human trials of APRV do demonstrate more modest outcomes for APRV when compared to LTV ventilation and are more consistent with our results [39–41]. When combined with the application of an adaptive and responsive ventilatory protocol, as would be experienced by human casualties in intensive care, rather than a fixed ventilatory pattern typically seen in animal modelling, the results for APRV reported here seem both credible and promising.

The study has a number of limitations. The size of the study is limited to 13 patients, constrained by the availability of adequate clinical datasets. This represents the best data currently available and facilitates our observational and iterative study in which we do not seek to offer outcomes of statistical significance. The casualties are all young adult males with no prior physical co-morbidity and no other injuries. This should be considered when extrapolating the results described to the wider population. The pharmacological effects of the sedative drugs that would be required to facilitate mechanical ventilation are not modelled. As a result, our in silico casualties have an artificially normal native mean arterial blood pressure and cardiac output. To attenuate the effects of confounding factors, we further assume that the patients are fully sedated, autonomic reflex modules are not enabled and patient ventilation is fully determined by the ventilator, potentially reducing the impact on indices of VILI [42]. Although the extensive computational model allows for the calculations of surrogate markers of VILI such as mechanical power and driving pressure, these need to be supported by direct histological evidence from clinical studies. Additionally, no changes were made to the underlying pathological insult suffered by each casualty over the 24 h of ventilation in which this study occurs.

## Conclusions

The results of this study suggest that PBLI casualties will respond well to either of the two modes of mechanical ventilation considered. APRV achieved the specified ventilatory targets with a lower PF ratio, sub-injury threshold levels of mechanical power, and lower levels of extravascular lung water compared to conventional LTV. Use of APRV may therefore offer casualties with PBLI important mortality-related benefits.

## Data Availability

All data generated and/or analysed during this study are included in this published article [and its supplementary information files].

## Abbreviations

APRV: Airway pressure release ventilation
ARDS: Acute respiratory distress syndrome
CO: Cardiac output [ml min^− 1^]
EELV: End expiratory lung volume [ml]
EVLW: Extravascular lung water [ml]
FiO_2_: Fraction of inspired air constituting of oxygen
I:E ratio: Ratio of inspiration time-expiration time
ICU: Intensive care unit
LTV: Low tidal volume ventilation; a lung protective mechanical ventilation strategy
MAP: Mean arterial pressure [mmHg]
*MP*: Mechanical power [J/min]
MPAP: Mean pulmonary arterial pressure [mmHg]
P_High_: Applied ‘high’ value of continuous positive pressure [cmH_2_O]
P_Low_: Applied ‘low’ value of continuous positive pressure [cmH_2_O]
PaCO_2_: Partial pressure of carbon dioxide in arterial compartment [kPa]
PaO_2_: Partial pressure of oxygen in arterial compartment [kPa]
PBLI: Primary blast lung injury
*PEEPt*: Total positive end expiratory pressure [cmH_2_O]
PF ratio: Ratio of arterial pressure of oxygen to fraction of oxygen in inspired air
*P*_*peak*_: Peak inspiratory pressure [cmH_2_O]
*P*_*plat*_: Plateau pressure [cmH_2_O]
T_High_: Duration of P high [s]
T_Low_: Duration of P low [s]
VILI: Ventilator-induced lung injury
*VR*: Respiration rate set by the ventilator [breaths per minute, bpm]
*V*_*T*_: Ventilator-delivered tidal volume [ml]
Δ*P*: Airway driving pressure, difference between plateau pressure and total PEEP [cmH_2_O]
